# Robotic-assisted laparoscopy is a feasible method for resection of deep infiltrating endometriosis, especially in the rectosigmoid area

**DOI:** 10.1177/03000605211032788

**Published:** 2021-08-19

**Authors:** Janika Hiltunen, Marja-Liisa Eloranta, Auni Lindgren, Leea Keski-Nisula, Maarit Anttila, Hanna Sallinen

**Affiliations:** 1 Department of Gynecology and Obstetrics, 60650 Kuopio University Hospital, Kuopio University Hospital, Kuopio, Finland; 2 Department of Health Sciences, Clinical Medicine, University of Eastern Finland, Kuopio, Finland

**Keywords:** Endometriosis, robotic-assisted laparoscopy, pain, rectosigmoid, quality of life, bowel operation

## Abstract

**Objective:**

This study aimed to compare outcomes of mini-invasive surgical treatment of endometriosis, especially conventional laparoscopy with robotic-assisted laparoscopy, and to evaluate the quality of life.

**Methods:**

One hundred three consecutive patients with endometriosis who had surgery from 2014 to 2017 owing to an indication of pain were enrolled in this retrospective study. The majority (n = 77, 75%) of patients underwent conventional laparoscopy and 18 (17%) had robotic-assisted laparoscopy. The quality of life was postoperatively assessed with a questionnaire.

**Results:**

The rates of parametrectomy (76% vs. 45%,) and rectovaginal resection (28% vs. 4%) were significantly higher in robotic-assisted laparoscopy than in laparoscopy. Additionally, the rate of bowel operations (50% vs. 17%), especially the shaving technique, was higher in robotic-assisted laparoscopy surgery than in laparoscopy (39% vs. 8%). There was no difference in the rate of postoperative complications between laparoscopy and robotic-assisted laparoscopy. Most (91%) of the patients who answered the questionnaire felt that surgical treatment had relieved their pain. In the laparoscopic and robotic-assisted groups, 88% of respondents felt that their quality of life had improved after surgery.

**Conclusions:**

This study suggests that robotic-assisted laparoscopy is a feasible method to resect deep infiltrating endometriosis, especially in the rectosigmoid area.

## Introduction

Endometriosis is an inflammatory, estrogen-dependent, chronic disorder in fertile-aged women. Endometriosis is defined as the presence of endometrial glands and stroma outside the uterine cavity. Although endometriosis is considered as a benign disease, it can cause severe chronic pain and infertility, and decrease the quality of life.^1^ Pharmacological treatments are the standard treatment for endometriosis.^2,3^ However, when deep infiltrating endometriosis (DIE) decreases the quality of life because of associated pain or due to dysfunction of the bowels, bladder or ovaries, then surgical treatment is necessary. Indications for surgical management are failure of medical management, the purpose of diagnosis, treatment of an adnexal mass or treatment of infertility.^4^

The mini-invasive approach of laparoscopic or robotic-assisted laparoscopy is highly recommended for endometriosis.^5^ However, a disadvantage of surgery is that when removing DIE lesions, complications often occur affecting gastrointestinal, urinary or sexual functions. Complications after surgery of DIE include rectal fistula (0.3%–2%), bowel stenosis (2%) and bladder atony (4%–6%).^6–8^ Therefore, the decision of surgery with its risks, benefits and extension should be carefully considered and discussed with patients who have endometriosis.

Currently, even extensive radical operations of the bowels or urinary tract can be performed mini-invasively.^9,10^ A few studies compared laparoscopic or robotic-assisted approaches in the surgical management of endometriosis.^11–15^ Robotic-assisted laparoscopic surgery is associated with a longer operation time than laparoscopic surgery,^12,16^ but results are controversial.^11,14^ The results of previous studies regarding benefits of robotic-assisted laparoscopy over conventional laparoscopy are somewhat heterogeneous. However, patients with features of a complex pelvic situation, such as severe endometriosis, an increased body mass index or prior surgeries, might benefit from robotic-assisted surgery.^17^

In our institution, robotic-assisted surgeries were initiated in 2016. This study aimed to evaluate the results of mini-invasive surgery for DIE in a single tertiary institution. Specifically, we aimed to 1) compare outcomes after conventional or robotic-assisted laparoscopic surgery in our institution and 2) evaluate the quality of life after surgery by a specific questionnaire.

## Methods

This retrospective study investigated consecutive patients who had been operated on for endometriosis-related pain between January 2014 and December 2017 in Kuopio University Hospital. The Research Ethical Committee of Northern Savo approved the study protocol (1012/13.02.00/2018) and written informed consent was obtained from all patients.

Endometriosis was diagnosed by laparoscopy or histologically in all patients. The stage of endometriosis was classified in accordance with revised American Society for Reproductive Medicine classification.^18^ Briefly, the stage of endometriosis is divided into the four stages of I (minimal), II (mild), III (moderate) and IV (severe). Data collected from medical files included prognostic, diagnostic and operative information, such as age, body mass index, operation date, preoperative symptoms, cancer antigen 125 (CA125) and human epididymis protein 4 (HE4) biomarkers, magnetic resonance imaging (MRI) findings, previous operations due to endometriosis, Clavien–Dindo classification,^19^ operation technique, operative areas, hormonal treatments and postoperative contact with a clinic because of pain from endometriosis. The upper normal limit for CA125 levels is 35 kU/L and that for HE4 levels is 70 pmol/L in premenopausal women in our hospital laboratory.

All of the patients were also sent a questionnaire inquiring about their well-being in January 2019. This questionnaire included questions about pain postoperatively, if the operation caused any short- or long-term harm, alternative treatments they had tried and their benefits, whether and how endometriosis was still affecting their daily lives and if the patients felt that the operation was beneficial and caused some change in their quality of life. The numeric rating scale (NRS) from 0 to 10 was used, where 0 indicates no pain and 10 the worst possible pain.

IBM SPSS Statistics for Windows, version 27 (IBM Corp., Armonk, NY, USA) was used in statistical analysis. Values are presented as mean ± standard deviation, unless otherwise stated. The Kruskal–Wallis test followed by the Mann–Whitney test for continuous variables in multiple comparisons were used when appropriate. We used the chi-square test to analyze frequency tables. A p value of < 0.05 was considered significant.

## Results

### Preoperative symptoms and treatments

The characteristics of the patients are shown in Table 1. All patients (n = 103) enrolled in this study experienced symptoms of pain. The most common symptoms were pelvic pain, followed by dyspareunia and dyschezia. Two patients reported shoulder pinch. The symptoms were continuous in more than half of the patients, symptoms occurred mainly during menstruation in approximately 40% and symptoms were only experienced occasionally in the remaining 5% (Table 1).

**Table 1.** Clinicopathological characteristics of patients with endometriosis (n = 103)

Variables	
Median age at the operation, years	37 (range: 17–55)
Median BMI, kg/m^2^	25 (range: 18–39)
Preoperative symptoms, n (%)	
Pelvic pain	102 (99)
Dyspareunia	51 (50)
Dyschezia	43 (42)
Dysuria	20 (20)
Vibration pain	21 (20)
Shoulder pinch	2 (2)
Frequency of preoperative symptoms, n (%)	
Occasional	5 (5)
Limited to menstruation	40 (39)
Constant	58 (56)
Preoperative imaging and biomarkers, n (%)	
MRI	73 (71)
Median CA125	57 (range: 7–535)
Median HE4	33 (range: 20–62)
Hormonal treatments preoperatively, n (%)	
Progesterone	52 (51)
Combined oral contraceptives	70 (68)
Hormonal IUD	40 (39)
GnHR agonistaromatasein	15 (15)
Aromatase inhibitor	5 (5)
Operative techniques, n (%)	
Laparoscopy	76 (75)
Robotic-assisted laparoscopy	18 (17)
Laparotomy	3 (3)
Vaginal hysterectomy	5 (5)
Endometriotic scar tissue removal	1 (1)

BMI, body mass index; MRI, magnetic resonance imaging; CA125, cancer antigen 125; HE4, human epididymis protein 4; IUD, intrauterine device; GnHR, gonadotrophin-releasing hormone.

Preoperative hormonal treatments are shown in Table 1. Two thirds of the patients used combined oral contraceptives, approximately half were receiving progesterone treatment, and 39% (n = 40) had a hormonal intrauterine device.

### Preoperative MRI and serum markers

As shown in Table 1, 73 (71%) patients underwent MRI before surgery to evaluate the presence and location of possible DIE. Forty-two (58%) patients showed signs of retrocervical DIE in MRI. The majority (71%) of patients who had evidence of retrocervical DIE in preoperative MRI underwent retrocervical resection of DIE in surgery (p = 0.011 vs patients who did not undergo retrocervical resection).

In the majority (n = 43, 72%) of patients with CA125 level measurement, preoperative serum CA125 levels were above the normal limit. In contrast, serum HE4 levels were within the normal limit (n = 37).

### Surgical techniques

Most patients underwent conventional laparoscopy or robotic-assisted laparoscopy (Table 1). In three patients, laparotomy was performed. The reasons for performing laparotomy were poor lung dysfunction in one patient and complex adhesions in the abdominal cavity in two patients. No conversions to laparotomy were undertaken. Additionally, five patients underwent vaginal hysterectomy because of endometriosis of the uterus, and in one patient, endometriotic tissue was removed from a cesarean section scar.

**Figure 1.**
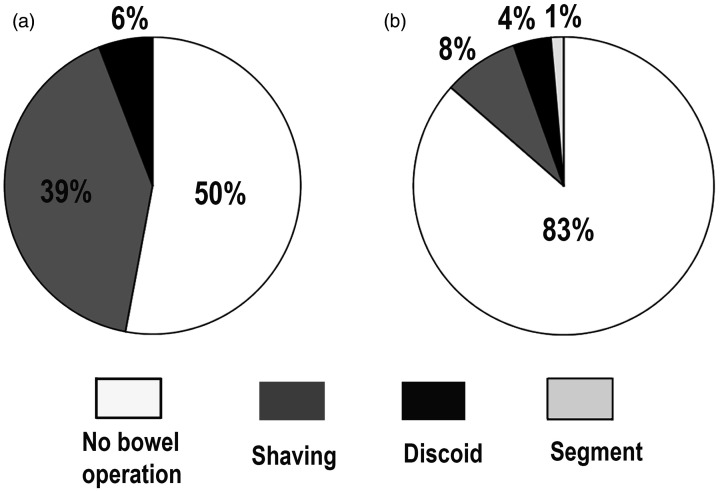
Pie charts showing the rates of bowel operations. Significantly more bowel operations were performed in robotic-assisted laparoscopy (a) than in conventional laparoscopy (b) (p = 0.011). Shaving was used significantly more often in robotic-assisted laparoscopy (a) than in conventional laparoscopy (b) (p = 0.011).

Details of the operated areas are shown in Table 2. When we compared only conventional laparoscopy and robotic-assisted laparoscopy, significantly higher rates of parametrectomy (p = 0.036) and rectovaginal resections (p = 0.001) were performed in robotic-assisted laparoscopy than in conventional laparoscopy. Additionally, significantly more bowel operations were performed in robotic-assisted laparoscopy than in conventional laparoscopy (p = 0.011). In particular, the shaving technique was applied more frequently in robotic-assisted laparoscopy than in conventional laparoscopy (p = 0.011) (Figure 1).

**Table 2.** Surgical procedures and complications

Variables	Laparoscopy	Robotic	
	(n = 76)	(n = 18)	
	n (%)	n (%)	p value
Stage of endometriosis			ns
I, minimal	7 (9)	0	
II, mild	26 (34)	3 (17)	
III, moderate	28 (37)	11(61)	
IV, severe	15 (20)	4 (22)	
Hysterectomy	34 (45)	7 (39)	ns
Adnexectomy	27 (36)	5 (28)	ns
Unilateral	17 (22)	1 (1)	
Bilateral	10 (13)	4 (24)	
Endometrioma resection	36 (41)	9 (50)	ns
Unilateral	23 (30)	7 (41)	
Bilateral	8 (10)	2 (12)	
Retrocervical resection	35 (46)	11 (65)	ns
Rectovaginal resection	3 (4)	5 (28)	0.001
Peritoneal resection	40 (53)	14 (78)	ns
Parametrectomy (e.g., sacral ligament)	34 (45)	13 (76)	0.036
Bowel operations	13 (17)	9 (50)	0.011
Shaving	6 (8)	7 (39)	0.011
Discoid resection	3 (4)	1 (6)	ns
Segmental resection	1 (1)		ns
Appendectomy	2 (3)	1 (6)	ns
Bladder resection	1 (1)	1 (1)	ns
Ureter operation	0	0	ns
Diaphragm resection	4 (5)	0	ns
Scar tissue removal	1 (1)	0	ns
Deliberation of adhesions	50 (66)	16 (89)	ns
Intraoperative complications	0	1 (6)	ns
Postoperative complications			ns
Clavien–Dindo grade			
I	8 (11)	2 (11)	
II	17 (22)	2 (11)	
IIIa	0	1 (6)	
IIIb	0	1 (6)	
No complications	51 (67)	11 (61)	ns
Median BMI, kg/m^2^	26 (19–39)	24 (18–38)	ns
Patients with previous pelvic surgery (%)	21 (28)	7 (39)	ns
Postoperative hormonal treatment	40 (53)	11 (61)	ns
Postoperative visit to the clinic owing to pain	26 (34)	3 (17)	ns

Robotic, robotic-assisted laparoscopy; BMI, body mass index; ns, not significant.

### Complications

There were no significant differences in the rates of postoperative complications between conventional laparoscopy and robotic-assisted laparoscopy (Table 2). The most common postoperative complications were urinary and genital infections, prolonged pain and short-term dysuria. Two patients had more severe postoperative complications (Clavien–Dindo IIIa and IIIb), with an abscess in the pouch of Douglas in one patient and a rectovaginal fistula in one patient. Only one intraoperative complication was observed, which was perforation of the rectum. This perforation was sutured immediately during surgery and no postoperative symptoms due to perforation were observed.

### Postoperative questionnaire of well-being

Almost half (44%, n = 45) of the patients returned the well-being questionnaire, which was sent to them after the operation. The median time between their operation and their answers to the questionnaire was 38 months (range: 14–61 months). Detailed results of the questionnaire are shown in Table 3. The majority (n = 34, 76%) of the respondents had undergone conventional laparoscopy and nine (20%) had undergone robotic-assisted laparoscopy. Only one respondent had been treated with laparotomy. Laparotomy was excluded from this assessment because of the lack of answers from patients who had been treated with laparotomy.

**Table 3.** Results of the well-being questionnaire postoperatively

Variables	Laparoscopy	Robotic
Number of patients who answered the questionnaire	34	9
Did surgical treatment relieve the pain?, n (%)		
Completely	13 (38)	5 (56)
Reduced pain considerably	14 (41)	2 (22)
Reduced pain quite a lot	4 (12)	2 (22)
No effect on pain	2 (6)	
Increased pain	1(3)	
Did surgical treatment cause adverse effects?, n (%)		
Shortly after surgery	18 (53)	5 (56)
Long-term dysuria	4 (12)	
Long-term dyschezia	2 (6)	
Long-term dyspareunia	1 (3)	
I think that the operation was useful, n (%)	30 (88)	9 (100)
What was your quality of life after the operation?, n (%)		
Improved	30 (88)	7 (88)
Stayed the same	3 (9)	1 (13)
Became worse	1(3)	
I currently have endometriosis-related pain, n (%)	21 (62)	3 (33)
I currently have endometriosis-related pain, n (%)		
Every day	2 (6)	
Weekly	2 (6)	1 (11)
Monthly	8 (24)	
Seldom	8 (24)	2 (22)
I currently have pain, n (%)		
Menstrual pain	12 (35)	2 (22)
Dyspareunia	11 (32)	1 (11)
Dyschezia	9 (27)	1 (11)
Dysuria	6 (18)	1 (11)
Vibration pain	6 (18)	1 (11)
Shoulder pinch	6 (18)	1(11)
How much does endometriosis affect your life currently?, n (%)		
Not at all	18 (53)	6 (67)
Sometimes	13 (38)	3 (33)
A lot	3 (9)	

Robotic, robotic-assisted laparoscopy.

Most (91%) of the respondents felt that surgical treatment had relieved their pain and 90% of the respondents thought that the operation had been beneficial. In the laparoscopic and robotic-assisted groups, 88% of the respondents felt that their quality of life had improved after surgery.

**Figure 2.**
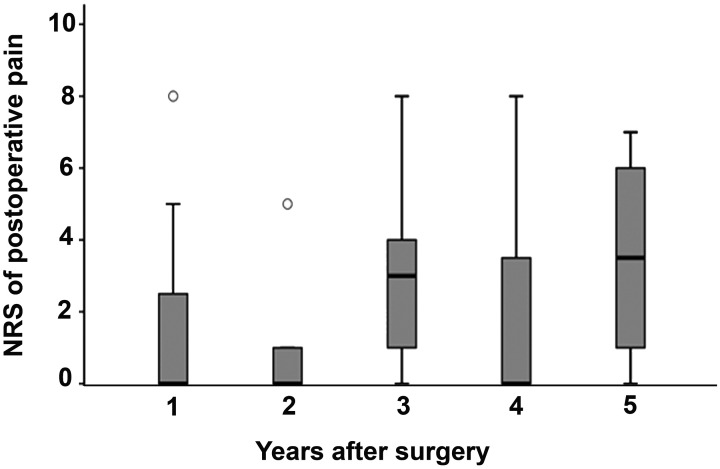
Numeric rating scale scores of postoperative pain after conventional laparoscopy or robotic-assisted laparoscopy. The first 2 years of follow-up included robotic-assisted laparoscopy and conventional laparoscopy. After this time, only conventional laparoscopy was included

Two-thirds (62%) of the respondents who had laparoscopy and one-third of respondents who had robotic-assisted laparoscopy reported that they were still experiencing pain due to endometriosis less or more often than monthly. Patients were asked in the questionnaire to score their pain by the NRS at the current moment. The mean NRS value was 1.9 ± 1.2 at 1 year after surgery, 1.0 ± 0.8 at 2 years, 3.1 ± 1.0 at 3 years, 1.7 ± 1 at 4 years and 3.6 ± 0.8 at 5 years (Figure 2). There were no significant changes in NRS values between time points or between laparoscopic or robotic-assisted surgery.

Approximately half of the respondents described short-term adverse effects after the operation (Table 3). Dysuria, pain, urinary and genital infections and catheterization were the most commonly reported adverse effects. Moreover fever, drip leakage of urine, tingling feelings in the uterine area and less intense orgasms were reported in the questionnaires. The rate of short-term adverse effects was similar in patients who had conventional laparoscopy to those who had robotic-assisted laparoscopy. Long-term dyschezia was reported by two respondents and long-term dyspareunia by one respondent who had undergone conventional laparoscopy. Some respondents also described adhesion pain, pelvic pain, menopausal symptoms and neuralgia in the scar area.

## Discussion

We found that robotic-assisted laparoscopy was a feasible method for resection of DIE, especially in the rectosigmoid area. Furthermore, pain and quality of life of the patients were evaluated by asking them to fill in a questionnaire. Most of the responders reported that surgery relieved their endometriosis-related pain and their quality of life had improved.

We report a single tertiary center experience of mini-invasive surgical treatment of painful endometriosis. Our patients represent a typical cohort of those who have endometriosis-related pain.^13^ Pelvic pain, dyspareunia and dyschezia were the most common symptoms in our patients. Hormonal treatments were widely used preoperatively in most cases. According to the European Society of Urogenital Radiology, MRI is recommended as a second-line imaging technique preoperatively.^20^ Transvaginal ultrasound and MRI achieve a similar accuracy in the diagnosis of DIE.^21^ Currently, MRI imaging is a routine procedure before surgery to evaluate the location and extent of DIE being used in addition to transvaginal ultrasound in our hospital.

CA125 levels are often elevated in patients with endometriosis. As expected, in our cohort, 72% of the patients had elevated CA125 levels. However, the benign nature of these findings was confirmed because HE4 levels were normal in all of our patients and no ovarian carcinomas were diagnosed.

Kondo et al. reported complication rates in patients who underwent a rectal operation that involved segmental resection, discoid excision or shaving.^22^ They found that less complications were associated with shaving than with segmental resection. In a large study by Mabrouk et al., the overall rate of short-term postoperative complications was significantly higher in patients who underwent segmental resection compared with those who underwent discoid excision or shaving.^8^ Furthermore, segmental resection does not appear to achieve more long-lasting improvement of symptoms compared with discoid resection or shaving.^23^ Especially at the level of the low rectum, shaving is the recommended method to avoid injury of vascular and sympathetic and parasympathetic nerve bundles.^7,24,25^ However, discoid excision or segmental resection is still an option to treat DIE at or above the sigmoid colon.^23^ In our study, we preferred shaving in accordance with recommendations. In the robotic-assisted laparoscopic group, shaving was used in 78% of patients who had undergone a bowel operation and no segmental resection was performed. The management of bowel endometriosis depends on the number of lesions, and their depth of invasion, size and circumferential involvement.^17,26^ Therefore, selection of the surgical technique needs to be tailored to each individual patient.

To date, there are only limited data comparing management of rectosigmoid DIE between robotic-assisted laparoscopy and conventional laparoscopy. The LAROSE trial, which was a randomized, multicenter trial, compared the treatment of endometriosis between robotic-assisted laparoscopy and conventional laparoscopy.^11^ This trial was not able to detect any differences in perioperative outcomes or the operative time between the robotic-assisted procedure and laparoscopy. Nonetheless, patients who required bowel resection were excluded from this trial. Results from other smaller mainly retrospective studies were heterogeneous. Some of these studies reported longer operation times with robotic-assisted procedures than with laparoscopy, while other studies found benefits from robotic-assisted surgery.^12,14,16^ Ercoli et al. showed that robotic-assisted laparoscopic nerve-sparing rectal nodulectomy appeared to be a feasible and safe approach in treating isolated retrocervical–rectal DIE.^27^ Recently, intravenous indocyanine green and near-infrared radiation imaging were reported to have an additional benefit in rectosigmoid endometriosis in assessing the blood supply of the bowel after resection.^28^ These techniques might also be helpful in separating endometrial nodules from healthy tissue. However, intraoperative near-infrared radiation imaging can be used during conventional or robotic-assisted laparoscopy.^29^

In our study, there was no difference in the rate of complications between patients who had robotic-assisted laparoscopy or conventional laparoscopy. However, two patients who had robotic-assisted laparoscopy had Clavien–Dindo grade III postoperative complications. This complication rate was acceptable because these patients had complicated DIE in the pelvis. Our results are also in line with a recent pilot study that compared robotic-assisted and conventional laparoscopy in treating colorectal endometriosis.^30^ In our cohort, all of the laparotomies were performed when robotic-assisted surgeries were not available in our institution. In the current study, no conversions to laparotomy were performed, which suggested the feasibility of using robotics. However, a multidisciplinary robotic team is necessary to operate on patients with rectosigmoid or urinary tract DIE.

Approximately 50% to 80% of patients with endometriosis consider a surgical treatment to be beneficial for endometriosis-related pain during the first 2 years after the operation.^31,32^ However, after 2 to 5 years, 36% of surgically treated patients might need to undergo a new operation^33^ These results are in line with the present findings. In the present study, most of the patients with endometriosis-related pain reported less pain and an improvement in their quality of life after surgery. According to the NRS scores, during the first 2 years after surgery, the patients’ pain symptoms were less intense, but subsequently, a trend towards higher NRS scores was observed. Notably, in this study, the first 2 years of follow-up included patients who had undergone either robotic-assisted or laparoscopic surgery, but the later evaluation included only those who had been treated with conventional laparoscopic operations.

There are some limitations to this study. First, our study was retrospective and the number of patients was limited. There might have been some bias because the more complex cases were routinely operated on using robotic-assisted techniques after 2016. These patients had a shorter follow-up time than patients who were operated on before this time. Second, our questionnaire of well-being has not been validated. Third, we had no information on the quality of life before the patients had surgery. Furthermore, pain was the only symptom that we evaluated.

In conclusion, the present study suggests that robotic-assisted laparoscopy is a feasible method to resect DIE. Mini-invasive surgical treatment also improves the quality of life in the majority of patients suffering from endometriosis-related pain. Further prospective investigations of mini-invasive treatment of patients with bowel endometriosis are warranted.
